# Supplementary figures and images for: Targeted Reactivation of FMR1 Transcription in Fragile X Syndrome Embryonic Stem Cells
Source: Front Mol Neurosci. 2018 Aug 15;11:282. doi: 10.3389/fnmol.2018.00282 (PMC6104480; doi:10.3389/fnmol.2018.00282)

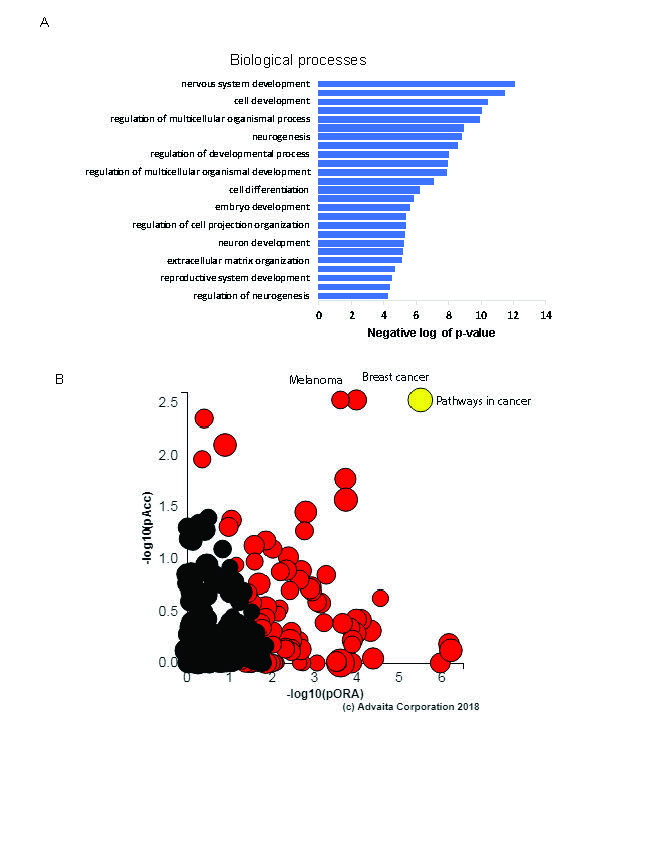

Supplement: FIGURE S1 — Gene ontology analysis and pathways for WT vs. late FXS hESCs. (A) Graph depicting top Gene Ontology (GO) terms for the RNA-seq data comparing WT and FXS hESCs. X-axis shows the GO terms and Y-axis shows the negative log (base 10) of the p-values for each category. (B) All pathways from the WT and FXS hESC RNA-seq analysis are plotted in terms of the two types of evidence computed by iPathwayGuide using Impact Analysis (Tarca et al., 2009): over-representation on the x-axis (pORA-Over Representation Analysis) and the total pathway accumulation on the y-axis (pAcc: Accumulated perturbation of the pathway). Each pathway is represented by a single dot, with significant pathways shown in red, non-significant in black. Both p-values are shown in terms of their negative log (base 10) values. Yellow dot represents cancer pathways. The adjacent red dots show pathways for melanoma and breast cancer. Figure obtained from iPathwayGuide (http://www.advaitabio.com/ipathwayguide). [file Image_1.JPEG]
